# Prolonged gastric mucosal haemorrhage following azacitidine administration in a patient with myelodysplastic syndrome

**DOI:** 10.1002/jha2.206

**Published:** 2021-05-01

**Authors:** Hiroaki Kitamura, Koichi Miyahara, Yasushi Kubota, Rika Tomimasu, Shinya Kimura, Masaharu Miyahara

**Affiliations:** ^1^ Department of Internal Medicine Karatsu Red Cross Hospital Karatsu Japan; ^2^ Division of Hematology, Respiratory Medicine and Oncology, Department of Internal Medicine, Faculty of Medicine Saga University Saga Japan

An 80‐year‐old man was referred to our hospital for further examination of abnormalities in a blood count. A full blood count showed thrombocytopenia and blasts were found in a peripheral blood film. Bone marrow aspiration revealed a mild hyperplasia with erythroid predominance, trilineage dysplasia, and the presence of 18.3% blasts. A diagnosis of myelodysplastic syndrome with excess blasts was made. He had received 75 mg/m^2^ subcutaneous azacitidine for 5 days in a 35‐day cycle. He had taken apixaban 2.5 mg twice daily for stroke prevention and vonoprazan fumarate 10 mg once daily. The patient had maintained stable disease by the administration of azacitidine.


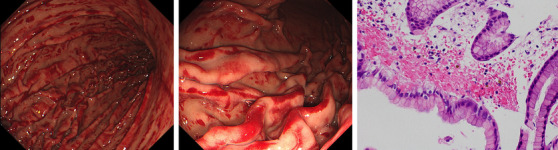


During 14th cycle of azacitidine, he complained with abdominal pain. Aluminium hydroxide gel and magnesium hydroxide relieved his symptom. However, it was exacerbated after the administration of azacitidine in each cycle. Anorexia was also accompanied. Physical examination of his abdomen showed no tenderness. Blood analysis showed the following: haemoglobin concentration, 128 g/L; white blood cell count, 3.8 × 10^9^/L (2% stab cells, 60% segmental cells, 30% lymphocytes, 5% monocytes, 1% eosinophils, and 2% basophils); platelet count, 91 × 10^9^/L; and C‐reactive protein, 1.2 mg/L at the start of 16th cycle. Upper gastrointestinal endoscopy was performed for further investigation on Day 4 in 16th cycle. Surprisingly, appearance of patchy and linear redness was expanded from gastric cardia to gastric lower body (left and middle). A gastric biopsy specimen demonstrated normal epithelium in gastric mucosa, and haemorrhage and haemosiderin deposition in the lamina propria (right). Because gastric mucosal haemorrhage following azacitidine was suspected, we discontinued administrating azacitidine while continuing to take apixaban and vonoprazan fumarate. Thereafter, the pain disappeared, and he regained his appetite. Although the endoscopic appearance remained unchanged after 1, 2, and 7 months of 16th cycle of azacitidine, it was almost completely disappeared after 21 months. Moreover, he has maintained stable disease after discontinuation of azacitidine.

Diffuse gastric mucosal haemorrhage is very rare. Azacitidine‐associated hemorrhagic event is suspected in the present case, but the mechanism of haemorrhage following azacitidine is still unknown. Besides, we consider that the cause of haemorrhage is not associated with neither thrombocytopenia due to azacitidine, direct oral anticoagulant nor other factors. Elucidation of the mechanism contributing to bleeding is important issue in patients managed by azacitidine.

## AUTHOR'S CONTRIBUTION

Conception and design: H. Kitamura, K. Miyahara, and Y. Kubota. Acquisition of data: H. Kitamura, K. Miyahara, R. Tomimasu, and M. Miyahara. Analysis of data: H. Kitamura and K. Miyahara.

Interpretation of data: H. Kitamura, K. Miyahara, Y. Kubota, S. Kimura, and M. Miyahara. Writing‐original draft: H. Kitamura, K. Miyahara, and Y. Kubota. Writing‐review and editing: H. Kitamura, K. Miyahara, Y. Kubota, R. Tomimasu, S. Kimura, and M. Miyahara.

## INFORMED CONSENT

The patient provided written informed consent.

## CONFLICT OF INTEREST

The authors declare no conflict of interest.

